# Colorimetric and Real-Time Loop-Mediated Isothermal Amplification (LAMP) for Detection of *Loa loa* DNA in Human Blood Samples

**DOI:** 10.3390/diagnostics12051079

**Published:** 2022-04-25

**Authors:** Begoña Febrer-Sendra, Pedro Fernández-Soto, Beatriz Crego-Vicente, Juan García-Bernalt Diego, Thuy-Huong Ta-Tang, Pedro Berzosa, Rufino Nguema, Policarpo Ncogo, María Romay-Barja, Zaida Herrador, Agustín Benito, Antonio Muro

**Affiliations:** 1 Infectious and Tropical Diseases Research Group (e-INTRO), Biomedical Research Institute of Salamanca-Research Centre for Tropical Diseases at the University of Salamanca (IBSAL-CIETUS), Faculty of Pharmacy, University of Salamanca, 37008 Salamanca, Spain; begofebrer@usal.es (B.F.-S.); beatrizcregovic@usal.es (B.C.-V.); juanbernalt95@usal.es (J.G.-B.D.); ama@usal.es (A.M.); 2 Malaria and Neglected Tropical Diseases Laboratory, National Centre of Tropical Medicine, CIBERINFEC, Institute of Health Carlos III, 28029 Madrid, Spain; tta@isciii.es (T.-H.T.-T.); pberzosa@isciii.es (P.B.); mromay@isciii.es (M.R.-B.); zherrador@isciii.es (Z.H.); abenito@isciii.es (A.B.); 3 National Control Programme of Onchocerciasis and Other Filariasis, Ministry of Health, Malabo, Equatorial Guinea; rufonguema@yahoo.es (R.N.); pncogo@psglobal.es (P.N.); 4 Fundación Estatal, Salud, Infancia y Bienestar Social, Institute of Health Carlos III (CSAI/ISCIII), 28029 Madrid, Spain

**Keywords:** *Loa loa*, loiasis, colorimetric LAMP, real-time LAMP, PCR, nested-PCR, dried blood spots, saponin/Chelex, microscopy, molecular diagnosis

## Abstract

Loiasis, caused by the filarial nematode *Loa loa*, is endemic in Central and West Africa. *Loa loa* has been associated with severe adverse reactions in high *Loa*-infected individuals receiving ivermectin during mass drug administration programs for the control of onchocerciasis and lymphatic filariasis. Diagnosis of loiasis still depends on microscopy in blood samples, but this is not effective for large-scale surveys. New diagnostics methods for loiasis are urgently needed. Previously, we developed a colorimetric high-sensitive and species-specific LAMP for *Loa loa* DNA detection. Here, we evaluate it in a set of 100 field-collected clinical samples stored as dried blood spots. In addition, *Loa loa*-LAMP was also evaluated in real-time testing and compared with microscopy and a specific PCR/nested PCR. A simple saponin/Chelex-based method was used to extract DNA. Colorimetric and real-time LAMP assays detected more samples with microscopy-confirmed *Loa loa* and *Loa loa*/*Mansonella perstans* mixed infections than PCR/nested-PCR. Samples with the highest *Loa loa* microfilariae counts were amplified faster in real-time LAMP assays. Our *Loa loa*-LAMP could be a promising molecular tool for the easy, rapid and accurate screening of patients for loiasis in endemic areas with low-resource settings. The real-time testing (feasible in a handheld device) could be very useful to rule out high-microfilariae loads in infected patients.

## 1. Introduction

*Loa loa* is a parasitic nematode that causes loiasis (commonly known as African eye worm). The parasite is transmitted to humans by Tabanid flies of the genus *Chrysops* and affects between 3 and 13 million people in the west and central regions of Africa [1]. Human loiasis is known to be endemic in eleven countries, including Angola, Chad, the Democratic Republic of the Congo, Cameroon, the Central African Republic, Equatorial Guinea, Ethiopia, Gabon, Nigeria, Republic of Congo and Sudan [2]. The main specific clinical manifestations include subcutaneous edema (Calabar swelling) and pruritus. Additionally, the ocular passage of the adult worm under the conjunctiva may be noticed. However, patients are usually asymptomatic or present nonspecific manifestations. Rarely, loiasis can cause damage in other organs [3]; although, a high *Loa loa* microfilaremia has been recently associated with an increased mortality risk [4]. Despite this, loiasis has been largely neglected as a public health problem in Africa and, even to date, the disease does not appear on the World Health Organization’s (WHO) list of neglected tropical diseases (NTD) [2]. Loiasis actually appear in the WHO-ESPEN (Expanded Special Project for Elimination of NTDs) program [5]. In addition, in some parts of sub-Saharan Africa, co-infections of *Loa loa* with other filarial species such as *Mansonella perstans* are possible [6,7]. *M. perstans* is considered the most common of the mansonellosis parasites (tiny fly-borne filarial nematodes), affecting probably more than 100 million people and with 600 million people living at risk of infection in Africa alone [8,9,10]. The clinical manifestations of mansonellosis are highly non-specific and shared with other co-infections in affected people, often making its diagnosis go unnoticed [8].

In loiasis-endemic areas, the only diagnostic method is a microscopic examination of a mid-day capillary blood sample for morphological identification of the *Loa loa* parasite. Microscopy is time-consuming, labor-intensive, requires skilled laboratory personnel and examines a small amount of blood, making it variable in terms of sensitivity and impractical for mass screening [6]. In an attempt to overcome the problems of traditional microscopy, the LoaScope, a smartphone-based video-microscope has been extensively validated to quantify *Loa loa* mf in fingerstick blood without the need for sample processing or staining [11,12,13,14,15]. However, LoaScope is calibrated to detect high-density *Loa loa* microfilaremia, and values under 150 mf/mL are considered unreliable [13,15]. In addition, the specificity on other filariae such as *Mansonella* spp. have not been well studied, and because it detects moving parasites, it can only be performed with fresh blood samples. Thus, it is considered a useful tool to support mapping projects for ivermectin-based Mass Drug Administration (MDA) programs, not as a diagnostic method. An alternative is serological testing, but it has low specificity due to cross-reactivity in patients with other filarial or helminthic infections and does not differentiate between past and current infection or quantify microfilaremia [16,17]. More recently, a *Loa* Antibody Rapid Test, commercially available as a lateral flow assay (LFA) platform, has been evaluated for epidemiological studies and to support mapping projects for ivermectin-based MDA programs to eliminate onchocerciasis and lymphatic filariasis. Nevertheless, the test has not been approved for use in individual case management [18]. 

In the last years, a series of polymerase chain reaction (PCR)-based molecular methods, such as conventional PCR, nested-PCR, and real-time quantitative PCR (qPCR), have been developed to detect *Loa loa* DNA with high accuracy and more sensitivity than parasitological and serological methods [19,20,21]. However, these technologies are not generally available in low-resource clinical settings due to their technical complexity. A number of alternative isothermal amplification methods targeting nucleic acids have been developed that offer significant improvements over PCR-based methods [22]. One of the most widely adopted is the loop-mediated isothermal amplification (LAMP) assay [23]. LAMP works under isothermal conditions (demanding minimal infrastructure) and amplifies the target nucleic acid using DNA polymerases with strand-displacement activity using a minimum of four, and up to six, specially designed primers [23]. This unique method of nucleic acid amplification makes the LAMP technology, compared to PCR-based methods, more sensitive, more specific, faster, more cost-effective and permits easy end-product visualization of the reaction in the diagnostic scenario [24,25,26]. To date, LAMP technology has been described for the detection of several filarial parasites, including *Loa loa* [27,28,29], *Wuchereria bancrofti* [30], *Brugia* spp. [31], *Onchocerca volvulus* [32,33], and *Mansonella perstans/ozzardi* [34].

On the other hand, it is important to note that venous blood sample collection for molecular analysis usually requires the support of health services and electricity for freeze-storage and delivery to laboratories, and this is particularly lacking in low-resource loiasis endemic areas. Capillary blood collection onto filter paper, known as dried blood spots (DBS), is a cheaper, more practical and convenient method to overcome these limitations [35] and makes it attractive for sample collection, storage and transportation from field settings [36]. It would be also desirable if these practical advantages could be accompanied by a reliable, sensitive and cost-effective method of nucleic acid extraction [37]. Several studies have already demonstrated the efficacy of using PCR-based methods and LAMP assays for the detection of infectious agents—including parasites, viruses and bacteria—in combination with simple DNA extraction methods from DBS [35,38,39]. Regarding this, Chelex-100 resin-based DNA extraction methods are among the most widely used because these procedures are simple, rapid, economic, involve no organic solvents and do not require multiple tube transfers avoiding excessive handling [2,38,40,41,42,43].

In a previous work, we developed an in-house high-sensitive and species-specific colorimetric LAMP assay for *Loa loa* DNA detection. This LAMP was successfully evaluated in the laboratory in human venous blood samples artificially contaminated with genomic DNA extracted from a *Loa loa* adult worm [27]. Here, for the first time, we evaluate our *Loa loa*-LAMP in a set of field-collected clinical samples, long-term stored as DBS. Additionally, it was the first time that the *Loa loa*-LAMP has been evaluated in real-time testing. The efficacy of both *Loa loa*-LAMP assays was compared with microscopy, as a reference diagnostic method, and with a specific PCR/nested-PCR assay [44]. 

## 2. Materials and Methods

### 2.1. Ethics Statement

Samples were obtained from the Laboratory of the National Centre of Tropical Medicine’s repository (Collection number C.0005278/ISCIII/Spain), Institute of Health Carlos III, Madrid, Spain, and registered according to the Spanish Law RD 1716/2011; article 22.1. Samples belonged to the malaria project PREMAVAL, to provide baseline data on malaria prevalence in Equatorial Guinea. The study was approved at the time by the Minister of Health and Social Welfare of Equatorial Guinea (MINSABS) and the Ethics Committee of the Spanish National Health Institute, Carlos III (CEI PI 22_2013-v3). The study was conducted in accordance with the Declaration of Helsinki. Informed consent was obtained from caregivers interviewed, the heads of the households and all subjects involved in the study.

### 2.2. Samples Obtaining and Selection

A set of 100 clinical samples were selected from a collection of DBS collected 7-years ago in a field survey carried out in Equatorial Guinea. The samples were stored at the Laboratory of the National Centre of Tropical Medicine, Institute of Health Carlos III, Madrid, Spain. When these samples were collected, thick (20 µL) and thin (5 µL) blood smears were first stained with 10% Giemsa solution and examined by experienced microscopists for the morphological identification of microfilariae (mf) according to published guidelines [45]. Microfilaremia was expressed as microfilariae per milliliter of blood (mf/mL) under 10× magnification and to determine the filarial species at 100× magnification with immersion oil. All fields were examined before declaring a slide negative. Mf densities were the average value found between the thick and thin slides by microscopic examination. A blood finger prick from each microscopically analyzed patient was spotted onto Whatman 903^TM^ paper (GE Healthcare Bio-Sciences Corp, Piscataway, NJ, USA) and stored in double zip-lock plastic bags with silica gel absorbent at −20 °C until analysis. The selected set of 100 DBS for this work (numbered from 1 to 100) was divided into 4 groups on basis of the microscopy findings, as follows: group 1 (*n* = 13) *Loa loa*-positive; group 2 (*n* = 11), *M. perstans*-positive; group 3 (*n* = 3), *Loa loa* and *M. perstans* mixed-positive; group 4 (*n* = 73), both *Loa loa* and *M. perstans*-negative. Table 1 shows the selected groups of DBS samples included in this study indicating mf counts.

### 2.3. DNA Extraction from Dried Blood Spots

DNA was extracted at the Laboratory of the National Centre of Tropical Medicine (CNMT, in Spanish), Institute of Health Carlos III, Madrid, Spain, via the Chelex-based method in combination with saponin following the protocol previously described by Plowe et al. [46] with slight modifications. Briefly, two filter paper discs of 5 mm in diameter were punched from each DBS using a handheld paper punch and placed into a 1.5 mL tube. Subsequently, 1 mL of 0.5% saponin (Fluka Biochemika, Sigma-Aldrich Chemie GmbH, Darmstadt, Germany) in autoclaved phosphate-buffered saline (PBS) 1× was added, mixed thoroughly, and incubated overnight at 4 °C or 37 °C for 1 h. After incubation, the brown solutions formed were aspirated and replaced with 1 mL of PBS 1×, and the tubes were incubated at 4 °C for an additional 30 min. Meanwhile, 100 mL of 5% Chelex 100 solution (Bio Rad, Richmond, CA, USA) in water was heated at 100 °C in a magnetic stirrer. The PBS was removed from tubes and 200 µL of heated 5% Chelex 100 were added to the paper discs, vortexed for 30 s, and placed in a heat-block at 98 °C for 10 min for incubation. After centrifugation at 13,000 rpm for 2 min, supernatants were recovered and centrifuged once again to remove any remaining Chelex 100 chelating resin before collection into clean tubes. Those extracted DNA samples were stored at −20 °C until further molecular analysis by PCR/nested-PCR at the laboratory of NCTM. Aliquots of 10 µL of each extracted DNA sample were sent frozen to the Center for Research in Tropical Diseases at the University of Salamanca (CIETUS), Salamanca, Spain, to perform molecular analysis by *Loa loa*-LAMP assay. 

### 2.4. Molecular Analysis

The 100 DBS selected in this study based on the microscopy results were analyzed in parallel for the species-specific molecular detection of *Loa loa* by a PCR/nested-PCR and both colorimetric and real-time LAMP assays following the methodology described below. 

#### 2.4.1. PCR/Nested-PCR Assay

A nested-PCR based on sequences of the repeat 3 region (15r3) of the gene encoding a *Loa loa* 15-kD protein was performed at Malaria and NTDs Laboratory-CNMT, Madrid, Spain, following the methodology described by Touré et al. (1998) [44] with slight modifications. Briefly, for initial PCR, 5 µL of template were amplified, and 2 µL of the first amplification product was used for the nested PCR. Amplification for initial PCR (product of 396 bp) was carried out using an Applied Biosystems GeneAmp^®^ PCR System 2700 for 30 cycles at 94 °C for 1 min (denaturation), 65 °C for 1 min (annealing), and 72 °C for 2 min (extension). For nested-PCR (product of 366 bp), amplification was carried out under the same conditions, but for 25 cycles. PCR products were analyzed by a QIAxcel Advanced automatic multicapillary electrophoresis system (QIAGEN GMBH, Hilden, Germany). For PCR reactions, positive controls consisted of *Loa loa*-microscopy positive clinical samples—first tested by filarial-real time qPCR and verified by sequencing following the methodology described by Ta-Tang et al. [21]. Negative controls consisted of filarial-microscopy negative clinical samples and *M. perstans*-microscopy positive samples from the CNMT collection. All positive results were confirmed in duplicates.

#### 2.4.2. Colorimetric LAMP Assay

LAMP assay based on an 839 bp *Loa loa*-specific repetitive DNA sequence (GenBank accession no.M34259.1) was performed at CIETUS, Salamanca, Spain, using the set of primers and reaction conditions previously described elsewhere by our group [27]. Briefly, LAMP reactions were carried out with a total of 15 μL reaction mixture containing 40 pmol of each FIP and BIP primers, 5 pmol of each F3 and B3 primers and 0.57 μL of *Bst* 2.0 Warm Start DNA polymerase (New England Biolabs Ltd., Hitchin, UK) with 1 μL of extracted DNA as a template. Reactions were incubated at 65 °C for 50 min in a heating block followed by heated at 80 °C for 5–10 min to stop the reaction. LAMP results were visually detected by color change (green: positive; orange: negative) by adding 1 μL of 1:10 diluted 10,000× concentration SYBR Green I (Invitrogen, Waltham, MA, USA) to the reaction tubes. When required, LAMP products were monitored using 1.5 % agarose gel electrophoresis and visualized under UV light in a transilluminator (UVP BioDoc-It^2^ Imager, Analytik Jena).

#### 2.4.3. Real-Time LAMP Assay

Real-time LAMP was performed with the same set of primers and reagents as the colorimetric LAMP, but with the addition of 0.24 μL/well of the DNA-binding dye EvaGreen 20× (BIOTIUM, San Francisco, CA, USA) to monitor the fluorescence in an Eco48 real-time PCR system (PCRmax, Beacon Road, Stone, Staffordshire, UK) programmed at 65 °C for 60 min followed by 10 min at 80 °C to stop the reaction by enzyme inactivation.

Genomic DNA (gDNA) from a *Loa loa* adult worm (0.5 ng/µL), and ultrapure water instead of the DNA template, were used as positive and negative controls, respectively, in all LAMP trials. All positive results were confirmed in duplicates.

### 2.5. Statistics

To estimate the accuracy of the molecular methods as diagnostic tests, the percentages of sensitivity, specificity, positive predictive value (PPV), negative predictive value (NPV) and kappa index for each molecular technique in comparison to microscopy as a reference diagnostic method were calculated using the free software WinEpi 2.0 [47]. The confidence intervals (CI) were established at 95%. To estimate and analyze the correlation between time to positivity (Tp) value in real-time LAMP positive assays and mf/mL a Pearson correlation test in R statistical software 3.6.3 version was used, creating a scatter plot using “ggplot2”, “ggpubr” and “ggrepel” packages.

## 3. Results

### 3.1. Application of Molecular Methods on Dried Blood Samples

#### 3.1.1. PCR/Nested-PCR Assay

Amplification products of the expected size (396 bp for initial PCR and/or 366 bp for nested-PCR) were obtained in 10/13 (76.92%) *Loa loa* microscopy-positive samples, and also in 2/3 (66.66%) *Loa loa* and *M. perstans* mixed-microscopy positive samples. No *M. perstans*-microscopy positive samples resulted positive for *Loa loa.* All *Loa loa* and *M. perstans*-negative samples resulted negative for *Loa loa* in PCR assays. Overall, 12/100 (12%) DBS were positive (see Table 1).

#### 3.1.2. Colorimetric LAMP Assay

**Figure 1:**
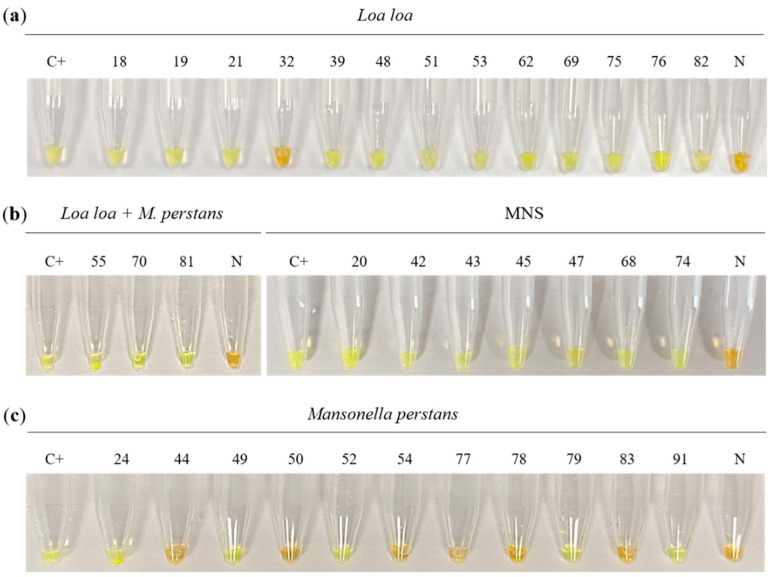
Examination of dried blood samples by conventional colorimetric *Loa loa*-LAMP. The figure shows the colorimetric visual detection of LAMP results in testing DBS using the SYBR Green I dye-based method: (**a**) *Loa loa, Loa loa*-microscopy positive samples. (**b**) *Loa loa* + *M. perstans*, *Loa loa* and *Mansonella perstans*-microscopy positive mixed samples; MNS, microscopy negative samples. (**c**) *Mansonella perstans*, *M. perstans*-microscopy positive samples. C+, *Loa loa* positive control (genomic DNA; 0.5 ng/µL); N, negative control (ultrapure water instead DNA template).

Visual detection of LAMP results in testing DBS using the SYBR Green I dye-based method is shown in Figure 1. LAMP detected 12/13 (92.31%) *Loa loa*-microscopy positive samples (Figure 1a). Only one sample (no. 32) was missed. In addition, 3/3 (100%) *Loa loa* and *M. perstans*-microscopy positive mixed samples and 7/73 (9.58%) microscopy negative samples resulted LAMP-positive (Figure 1b). Surprisingly, up to 5/11 (45.45%) *M. perstans*-microscopy positive samples resulted LAMP-positive for *Loa loa* (Figure 1c). In all, 27/100 (27%) DBS were LAMP-positive (see Table 1).

#### 3.1.3. Real-Time LAMP Assay

**Figure 2:**
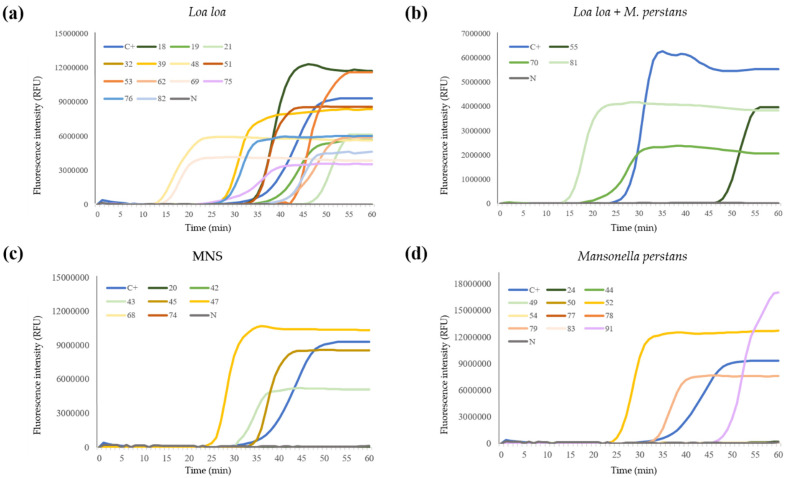
Detection of *Loa loa* in dried blood samples by real-time LAMP. The figure shows the results detected by fluorescence measured in relative fluorescence units (RFU) in a PCRmax Eco 48 Real-Time qPCR System: (**a**) *Loa loa*, *Loa loa*- microscopy positive samples. (**b**) *Loa Loa*+ *M. perstans*, *Loa loa* and *Mansonella perstans*-microscopy positive mixed samples. (**c**) MNS, microscopy negative samples. (**d**) *Mansonella perstans*, *M. perstans*-microscopy positive samples. C+, *Loa loa* positive control (genomic DNA;0.5 ng/µL); N, negative control (ultrapure water).

**Figure 3:**
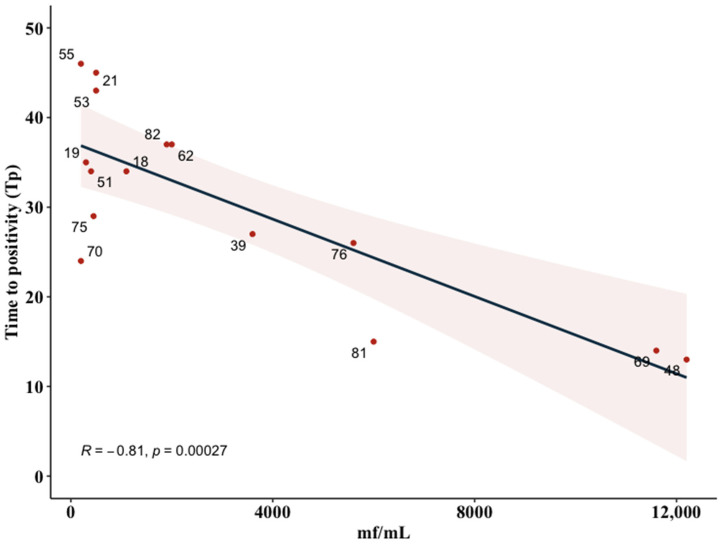
Correlation chart showing the relationship between time to positivity (Tp) values and microfilariae per milliliter (mf/mL) for the 16 samples microscopically positive for *Loa loa* that also tested positive for Loa loa in real-time LAMP assays. The numbers associated with the red dots indicate the number of the samples analyzed.

Detection of *Loa loa* in DBS by real-time LAMP is shown in Figure 2. A positive result was obtained in the same 12/13 (92.31%) *Loa loa*-microscopy positive samples than with the colorimetric LAMP assay (Figure 2a). As for visual LAMP detection, sample no. 32 was also missed by real-time LAMP. Moreover, 3/3 (100%) *Loa loa* and *M. perstans*-microscopy positive mixed samples (Figure 2b) and 3/73 (4.11%) microscopy negative samples resulted real-time LAMP positive (Figure 2c). Regarding the *M. perstans*-microscopy positive samples, 3/11 (27.27%) resulted positive (Figure 2d). In all, 21/27 (77.77%) DBS resulted in real-time LAMP positive (see Table 1). In addition, for the 16 samples *Loa loa*-microscopy positive (those included in group 1, except for sample no. 32, and those included in group 3) a relationship between Tp and. mf/mL values was established. The samples with the highest mf counts of *Loa loa* in microscopic examination (nos. 48, 69 and 81) were amplified with the shortest Tp values (Figure 3).

**Figure 4:**
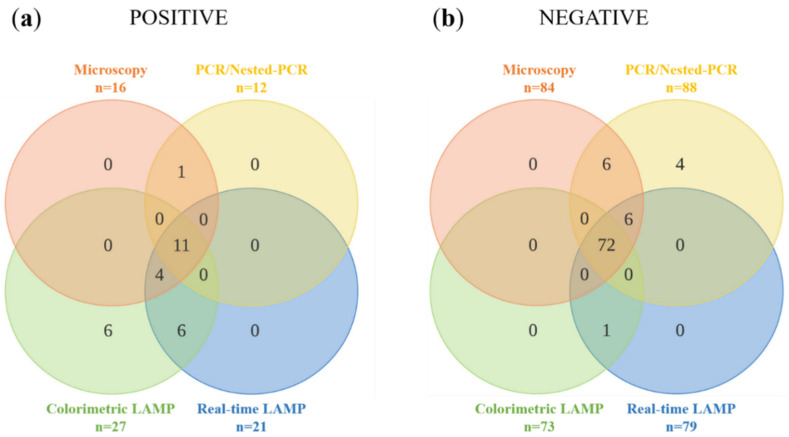
Venn diagrams for four-way comparison of microscopy, colorimetric LAMP, real-time LAMP and PCR/nested-PCR results in specific *Loa loa* detection: (**a**) Distribution of the samples with a *Loa loa* positive results for at least one test. (**b**) Distribution of the samples with a *Loa loa* negative result for at least one test.

The results of PCR/nested-PCR, colorimetric LAMP and real-time LAMP assays were compared with microscopy as the reference diagnostic method and the overlaps are shown using Venn diagrams in Figure 4. Up to 11 of the 16 DBS samples (68.75%) with microscopy-positive results were positive by the three molecular tests (Figure 4a). On the other hand, up to 72 of the 84 DBS samples (85.71%) with microscopy-negative were negative for all detection tests performed (Figure 4b). The estimation of the accuracy of the molecular methods applied as diagnostic tests for *Loa loa* detection is shown in Table 2. In summary, LAMP assays showed the highest sensitivity (94.1%) and the PCR/nested-PCR showed the best specificity (100%).

## 4. Discussion

Routine clinical diagnosis of human loiasis and mansonellosis depends on the detection of mf on Giemsa-stained thick and thin blood slides by microscopy based on morphological identification [6]. However, the efficacy of microscopic detection of mf is diminished by long pre-patency, periodicity in the case of *Loa loa,* and mild or occult infection without microfilaremia [48]. To solve these drawbacks, a number of molecular assays, both PCR-based and LAMP-based methods, have emerged in the last years to provide an accurate alternative for the identification and detection of filarial parasites. PCR-based methods, mainly real-time qPCR, are generally used in laboratories in developed countries, while LAMP-based methods are a cheaper and easy molecular option for low-income countries and have the potential for near point-of-care (POC) application [6].

In a previous work, our group developed an in-house colorimetric LAMP-based method for the specific detection of *Loa loa* DNA. This *Loa loa*-LAMP achieved a very high level of sensitivity, detecting up to 0.5 attograms (ag) of genomic DNA (which is equivalent to 1/1.000.000th of a mf) in fresh human venous blood samples artificially enriched with *Loa loa* DNA [27]. Now, in this work, we evaluate for the first time, our *Loa loa*-LAMP, both colorimetric and real-time testing, in a set of 100 clinical samples collected in a loiasis-endemic area long-term stored as DBS. The saponin/Chelex-based method was used to extract DNA from DBS for molecular analysis. This simple chelating resin-based procedure for DNA extraction was tried because it is cheap, fast and does not require multiple tube transfers, which avoids potential contamination when numerous samples need to be analyzed [40,49]. For example, the saponin/Chelex-100 DNA extraction method has already been widely and successfully used for the isolation of malaria parasite DNA from DBS for molecular analysis by PCR [42,43,50,51].

When analyzing DNA extracted from DBS, we obtained more LAMP-positive colorimetric and real-time results than PCR/nested-PCR results in samples from a group of patients with microscopy-confirmed infection by *Loa loa* (12/13; 92.30% vs. 10/13; 76.92%) and by *Loa loa* and *M. perstans* mixed infection (3/3; 100% vs. 2/3; 66.66%), respectively. Therefore, by using a simple method involving a chelating resin in combination with a highly sensitive molecular method, either a PCR-based or LAMP-based method, it is possible to detect *Loa loa* in DBS as a DNA source. However, LAMP assays showed a higher sensitivity than PCR/nested-PCR, as seems to be demonstrated by the fact that LAMP amplified all five *Loa loa*-positive samples with a lowest-microfilariae count between 300 and 500 mf/mL (nos. 19, 21, 51, 53 and 75) while PCR/nested-PCR only achieved amplification in two of these five samples (nos. 19 and 21). Unexpectedly, both colorimetric and real-time LAMP assays failed to amplify 1/13 samples (no. 32) with microscopy-confirmed infection by *Loa Loa* (with a high count of 2200 mf/mL), which was amplified by PCR/nested-PCR. Honestly, we do not know what could have caused this failure. A possible explanation for this could be associated with improper sample storage, repeated freezing and thawing cycles for different assays, or simple sample handling errors.

Furthermore, we obtained colorimetric LAMP-positive results for *Loa loa* in 5/11 (45.45%) samples (nos. 24, 49, 52, 79 and 91) with only microscopy-confirmed *M. perstans* infection. However, when performing the real-time LAMP assay, only 3/11 (27.27%) of those samples (nos. 52, 79 and 91) also resulted positive, but in two of them (nos. 24 and 49) no amplification was achieved. Interestingly, we also obtained colorimetric LAMP-positive results for *Loa loa* in 7/73 (9.58%) samples (nos. 20, 42, 43, 45, 47, 68 and 74) with microscopy-negative findings, but only 3/73 (4.11%) of those samples (nos. 43, 45 and 47) resulted positive by real-time LAMP assay. We sincerely believe that those colorimetric LAMP-positive results obtained for *Loa loa* in both *M. perstans* microscopy-positive samples and in samples with microscopy-negative findings could be true DNA *Loa loa* detection, since coinfections with *M. perstans* in co-endemic areas are very common [6,52], and it is also possible that they may have gone undetected under microscopy due to their well-known limited sensitivity for *Loa loa* [53]. Moreover, the higher sensitivity of our in-house LAMP over PCR/nested-PCR could also explain these positive results. The fact that in these two groups of samples (*M. perstans* microscopy-positive and microscopy-negative findings), up to six samples resulted positive by conventional colorimetric *Loa loa*-LAMP but not in real-time settings, could be explained because of the pre-amplified EvaGreen fluorescent dye in the reaction mixes for real-time monitoring. It has been demonstrated that EvaGreen is able to partially inhibit LAMP reactions by reducing both the rate and final levels of amplification [54]. On the other hand, it might be thought that the amplification of these samples by conventional colorimetric *Loa loa*-LAMP could have been non-specific amplification or post-amplification contamination by opening the tubes and adding SYBR Green I at the end of the reaction. However, we honestly believe that the specificity demonstrated by our *Loa loa*-LAMP in its previous development and setup (with no cross-reaction with *M. perstans* or *Brugia pahangi*, a filaria closely related to *Loa loa*) [27], the gentle handling of the tubes in a laminar flow hood, and the duplicate confirmation of positive results, rule out those possibilities.

Very interestingly, those samples with the highest mf counts of *Loa loa* on microscopic examination, including samples no. 48 (12,200 mf/mL) and no. 69 (11,600 mf/mL) in Group 1 (*Loa loa* microscopy-positive), and also sample no. 81 (6000 mf/mL) in Group 3 (*Loa loa* and *M. perstans* mixed infection), amplified with the shortest time to positivity (Tp) values when performing the real-time LAMP assay: 13, 14 and 15 min, respectively. Since serious adverse events can occur in individuals with high *Loa loa* mf loads due to ivermectin administration in onchocerciasis control and elimination programmes in Central Africa, and that the risk increases significantly when *Loa loa* loads exceed 8000 mf/mL [55], real-time *Loa loa*-LAMP could be very useful as a diagnostic tool to detect high mf loads (which would correlate to shorter Tp values) in infected individuals to prevent the risk of serious reactions. Considering that real-time *Loa loa*-LAMP can also be easily performed on a battery-powered handheld device for outdoor use (i.e., Genie III platform), and the simplicity of the saponin/Chelex-based DNA extraction from a small quantity of finger-prick blood samples stored as DBS, make this combination highly desirable as a promising tool applicable as a POC test for molecular diagnosis of loiasis in endemic areas with resource-limited settings. 

Finally, the LAMP-positive results detected in samples with negative microscopy results reinforce the idea that the sensitivity of our in-house *Loa loa*-LAMP assay (both the colorimetric and real-time) is also higher than that of microscopy for the detection of natural *Loa loa* infections in clinical samples. In this sense, our results are consistent with those obtained in a recent study in which LAMP is also found to be more sensitive than microscopy for the detection of experimental and natural *Loa loa* infections in *Chrysops* vectors [56]. Overall, statistically, the conventional colorimetric *Loa loa*-LAMP and real-time *Loa loa*-LAMP showed the highest sensitivity (94.1%) compared to microscopy; although, PPV for colorimetric *Loa loa*-LAMP was low (57.1%). In our experience in the development and application of LAMP technology for the diagnosis of parasitic infections, these statistical results (a high sensitivity and a low PPV) are also consistent with other studies comparing the accuracy of the LAMP assay with microscopy as a gold standard in different types of clinical specimens, either in urine (e.g., for urinary schistosomiasis diagnosis) [57] or feces (e.g., for amphimeriasis diagnosis) [58]. 

## 5. Conclusions

Considering the results obtained in this study when analyzing DBS samples, we can conclude that our conventional colorimetric in-house *Loa loa*-LAMP assay could be a promising molecular diagnostic tool for the easy, rapid, sensible and specific screening of patients for loiasis in endemic areas with low-resource settings. Moreover, the real-time *Loa loa*-LAMP testing (that can be easily performed in a handheld device with no electricity requirement), could be very useful to detect people with high mf loads to define more precisely which individuals and which communities are eligible to receive ivermectin in MDA against onchocerciasis and lymphatic filariasis in overlapping areas with *Loa loa*, thus reducing possible life-threatening complications. Further research would be desirable to further increase specificity (e.g., by including loop primers in the LAMP design, exploring other potential target *Loa loa*-specific DNA sequences) and develop a multiplex-LAMP that would allow for simultaneous detection of different filarial species in a single patient sample, especially in areas where loiasis is co-endemic with other filariasis.

## Figures and Tables

**Table 1:** Dried blood samples included in this study. Sample groups (G1, G2, G3, and G4), parasitological findings based on microscopy of thick and thin blood smears with Giemsa stain, sample numbers and microfilariae counts (expressed as microfilariae per milliliter of blood; mf/mL) are indicated. PCR/nested-PCR, colorimetric and real-time LAMP results obtained in this study are also included.

Sample Groups	Parasitological Finding	Sample Number	mf/mL	PCR/ Nested-PCR	Colorimetric LAMP	Real-Time LAMP
G1 (*n* = 13)	*Loa loa*	18	1100	+	+	+
		19	300	+	+	+
		21	500	+	+	+
		32	2200	+	-	-
		39	3600	+	+	+
		48	12,200	+	+	+
		51	400	-	+	+
		53	500	-	+	+
		62	2000	+	+	+
		69	11,600	+	+	+
		75	450	-	+	+
		76	5600	+	+	+
		82	1900	+	+	+
G2 (*n* = 11)	*Mansonella perstans*	24	200	-	+	-
		44	600	-	-	-
		49	800	-	+	-
		50	100	-	-	-
		52	100	-	+	+
		54	1300	-	-	-
		77	3200	-	-	-
		78	100	-	-	-
		79	400	-	+	+
		83	1000	-	-	-
		91	1000	-	+	+
G3 (*n* = 3)	*Loa loa/M. perstans*	55	200/200	-	+	+
		70	200/200	+	+	+
		81	6000/1500	+	+	+
G4 (*n* = 73)	No findings	20		-	+	-
		42		-	+	-
		43		-	+	+
		45		-	+	+
		47		-	+	+
		68		-	+	-
		74		-	+	-
		Remaining nos. up to 100		-	−66	−70

**Table 2:** Estimation of the accuracy of the molecular methods applied as diagnostic tests for *Loa loa*. Estimation of sensitivity, specificity, positive and negative predictive values and Kappa index by DNA amplification tests (colorimetric LAMP, real-time LAMP and PCR/nested-PCR) against microscopy as reference diagnostic method for current study for identifying *Loa loa* infection in the 100 patients’ dried blood samples analyzed. PPV, positive predictive value; NPV, negative predictive value; CI, confidence intervals; Kappa, Kappa index.

	Colorimetric LAMP	Real-Time LAMP	PCR/Nested-PCR
Sensitivity (95% CI)	94.1% (81.9–105.6%)	94.1% (82.9–105.3%)	75.0% (53.8–96.2%)
Specificity (95% CI)	87.5% (78.5–93.3%)	93.3% (88.2–98.5%)	100.0% (100.0–100.0%)
PPV (95% CI)	57.1% (36.8–74.3%)	72.7 % (54.1–91.3%)	100.0% (100.0–100.0%)
NPV (95% CI)	98.8% (96.0–101.3%)	98.8% (96.5–101.1%)	95.5% (91.1–99.8%)
Kappa (95% CI)	62.2% (43.6–80.7%) **	76.9% (57.6–96.2%) **	83.4% (67.5–99.3%) ***

** good agreement; *** excellent agreement.

## Data Availability

All data generated or analyzed during this study are included in this manuscript.
